# Supplementary material for: Retrosynthesis of multi-component metal−organic frameworks
Source: Nat Commun. 2018 Feb 23;9:808. doi: 10.1038/s41467-018-03102-5 (PMC5824804; doi:10.1038/s41467-018-03102-5)
Supplement: Supplementary file 1 — Supplementary Information [file 41467_2018_3102_MOESM1_ESM.pdf]

Supplementary Information

Retrosynthesis of Multi-Component Metal−Organic Frameworks

Yuan et al.

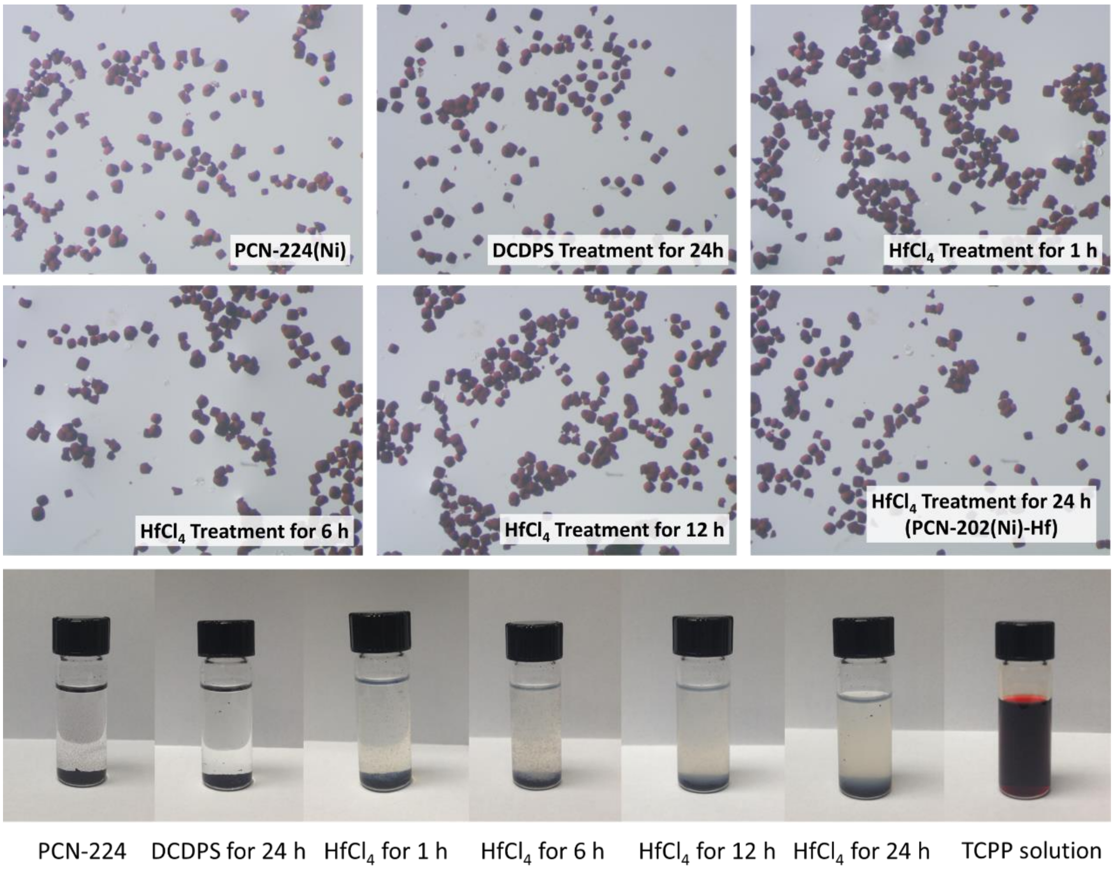

Supplementary Figure 1. Photos of crystals during the formation of PCN-202(Ni)-Hf.

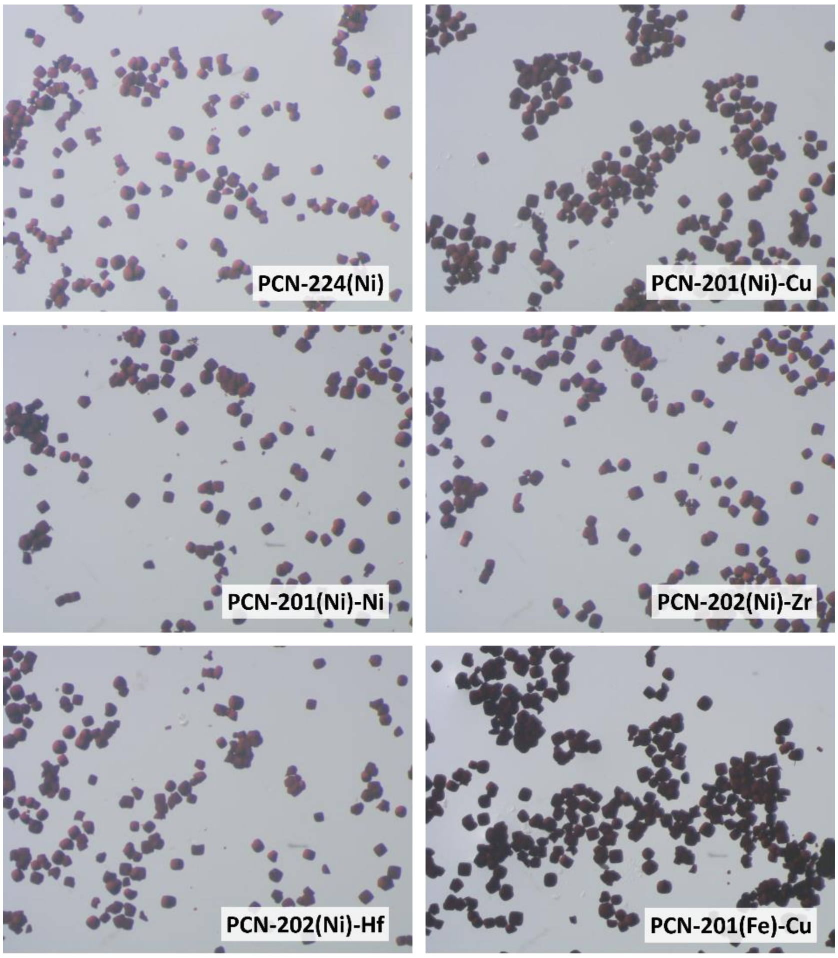

Supplementary Figure 2. Microscopic images of PCN-224(Ni), PCN-201(Ni)-Cu, PCN-201(Ni)-Ni, PCN-202(Ni)-Zr, PCN-202(Ni)-Hf, and PCN-201(Fe)-Cu.

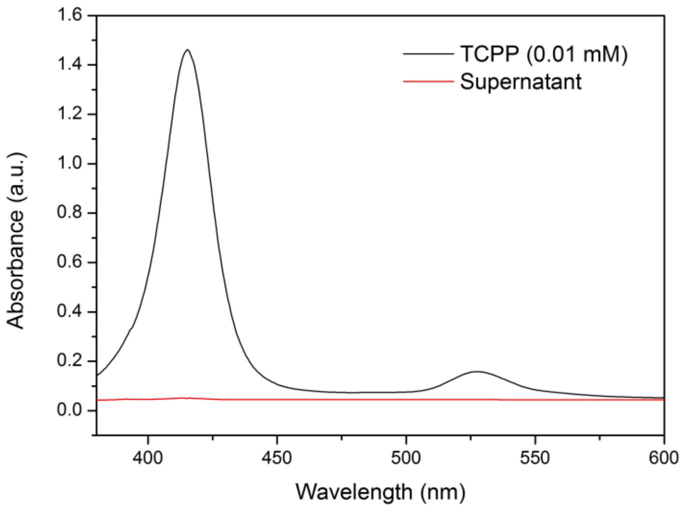

Supplementary Figure 3. UV spectrum of TCPP solution and the supernatant during the synthesis of PCN-202(Ni)-Zr.

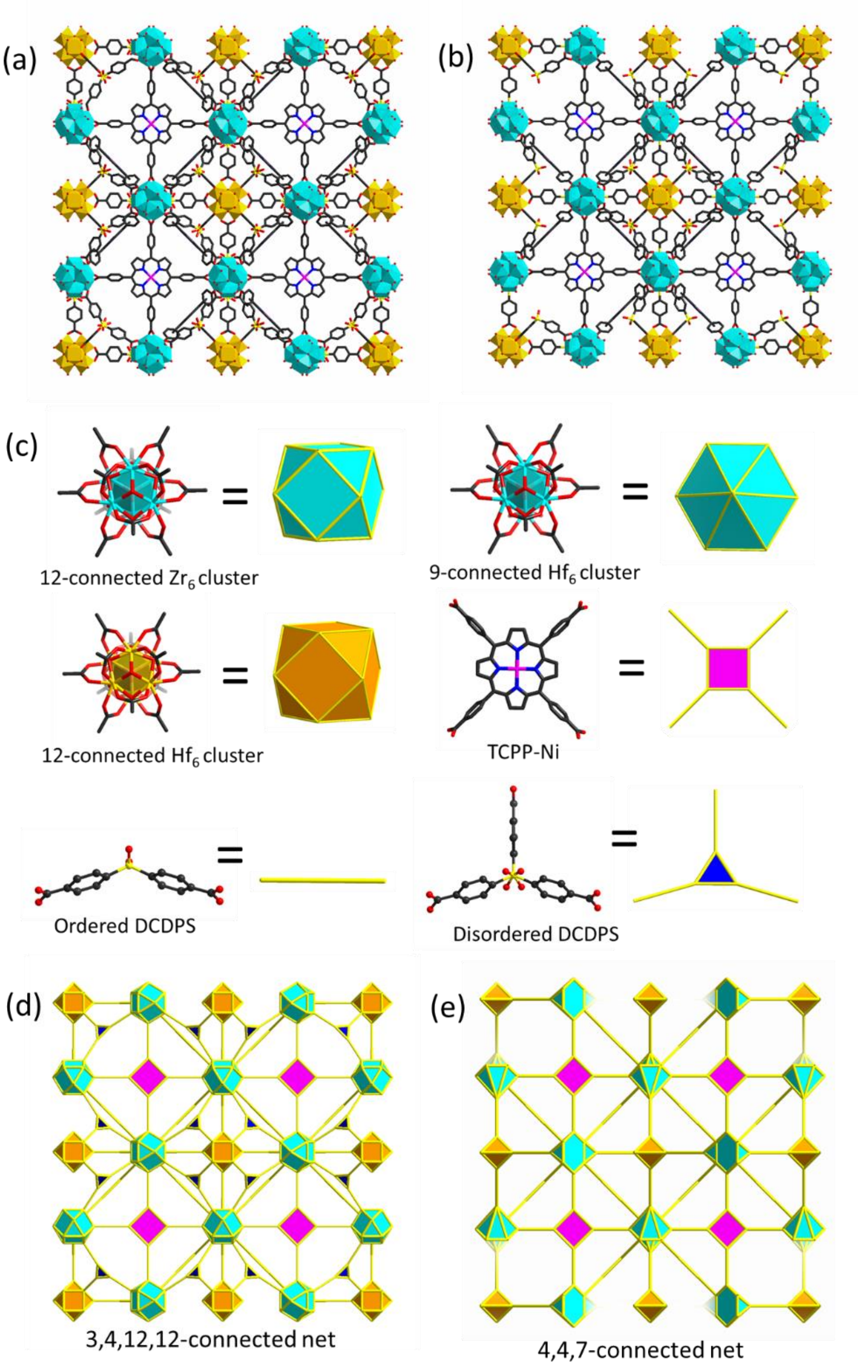

Supplementary Figure 4. (a) Crystal structure of PCN-202(Ni)-Hf with disorder. (b) simulated structure of PCN-202(Ni)-Hf without disorder. (c) Building units and their simplified topological elements. (d) and (e) topology of PCN-202(Ni)-Hf with and without disorder.

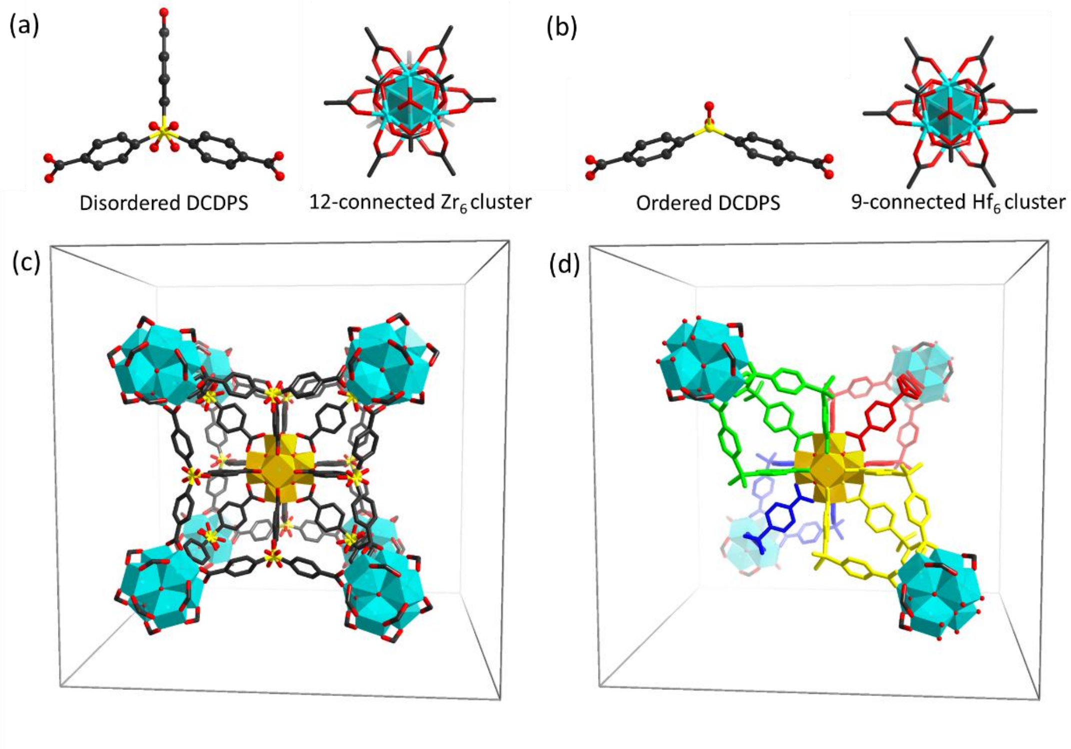

Supplementary Figure 5. (a) and (c) Crystal structure of PCN-202(Ni)-Hf with disorder. (b) and (d) simulated structure of PCN-202(Ni)-Hf without disorder.

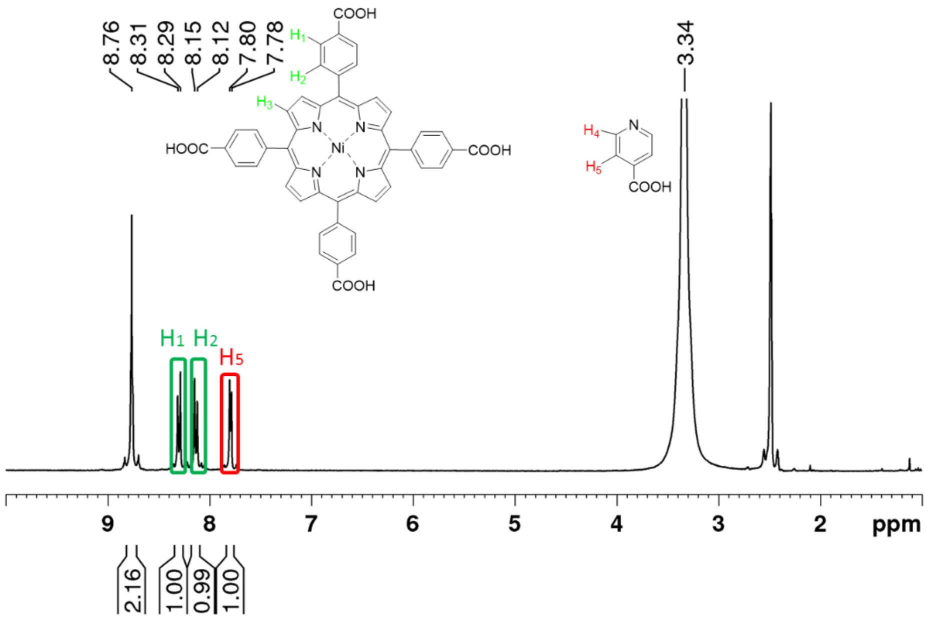

Supplementary Figure 6. ^{1}H NMR spectroscopy of digested PCN-201(Ni)-Cu**.**

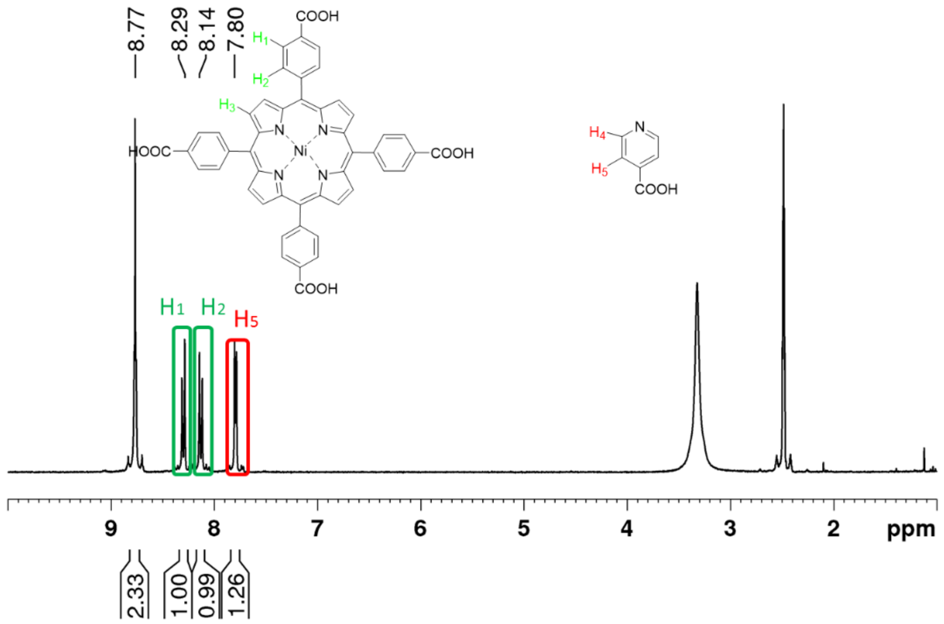

Supplementary Figure 7. ^{1}H NMR spectroscopy of digested PCN-201(Ni)-Ni**.**

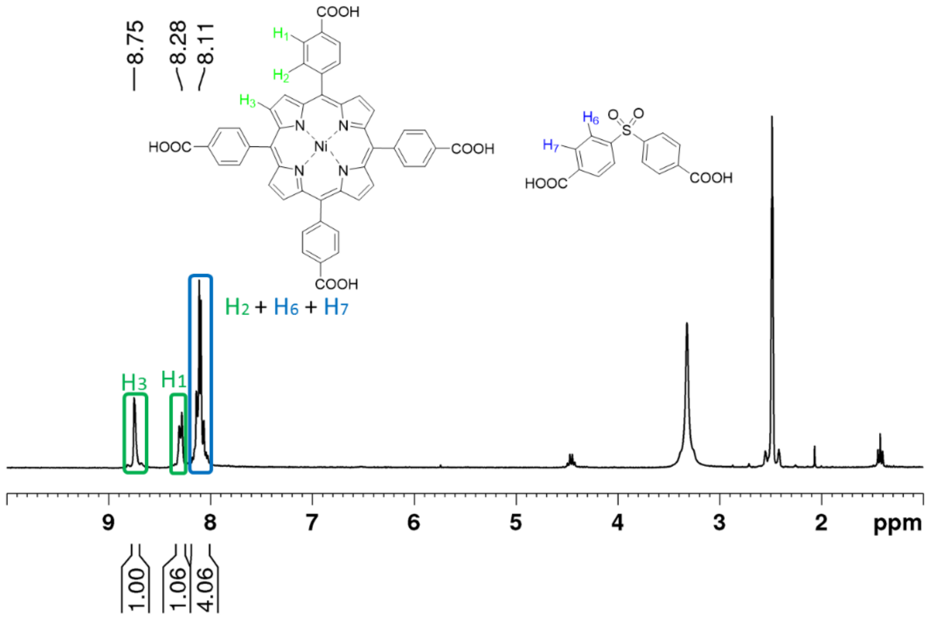

Supplementary Figure 8. ^{1}H NMR spectroscopy of digested PCN-202(Ni)-Hf**.**

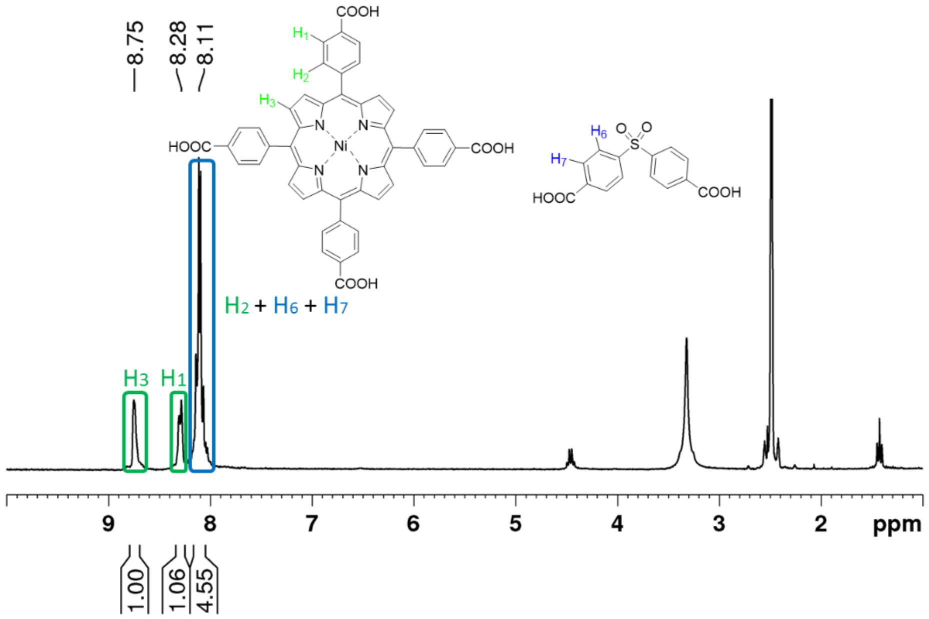

Supplementary Figure 9. ^{1}H NMR spectroscopy of digested PCN-202(Ni)-Zr**.**

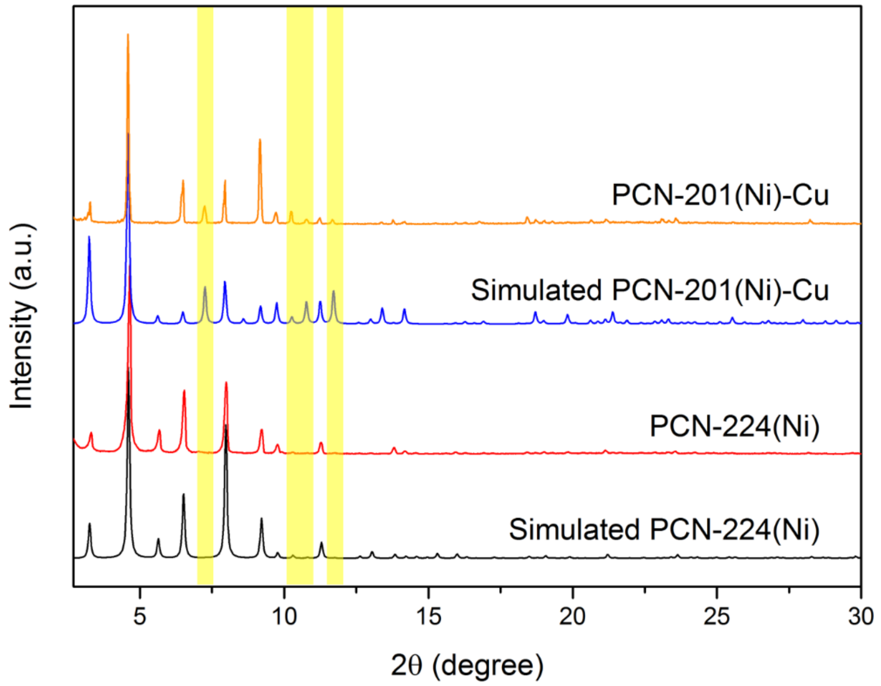

Supplementary Figure 10. A comparison of powder X-ray diffraction (PXRD) patterns for PCN-201(Ni)-Cu and PCN-224(Ni).

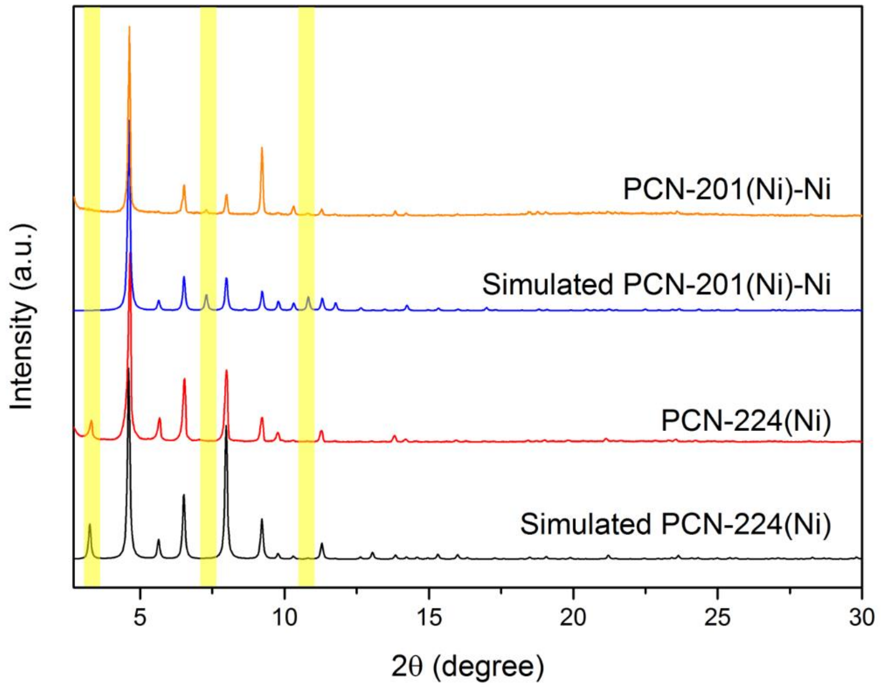

Supplementary Figure 11. A comparison of PXRD patterns for PCN-201(Ni)-Ni and PCN-224(Ni).

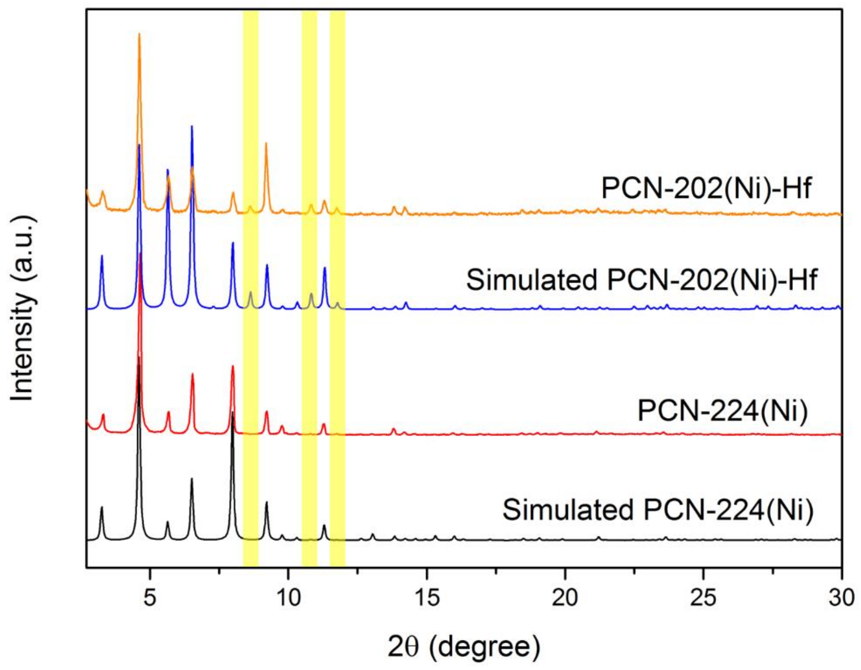

Supplementary Figure 12. A comparison of PXRD patterns for PCN-202(Ni)-Hf and PCN-224(Ni).

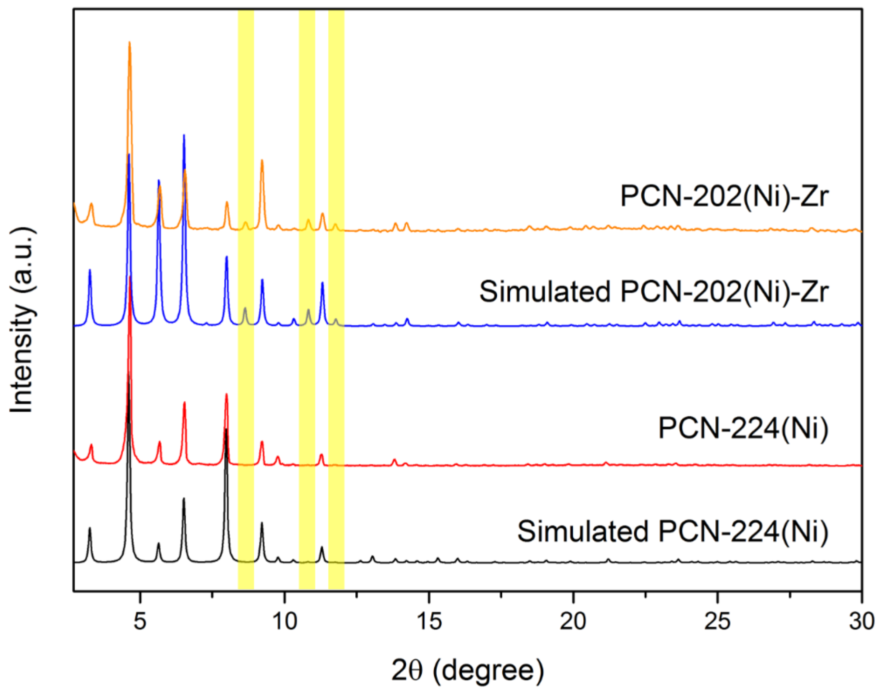

Supplementary Figure 13. A comparison of PXRD patterns for PCN-202(Ni)-Zr and PCN-224(Ni).

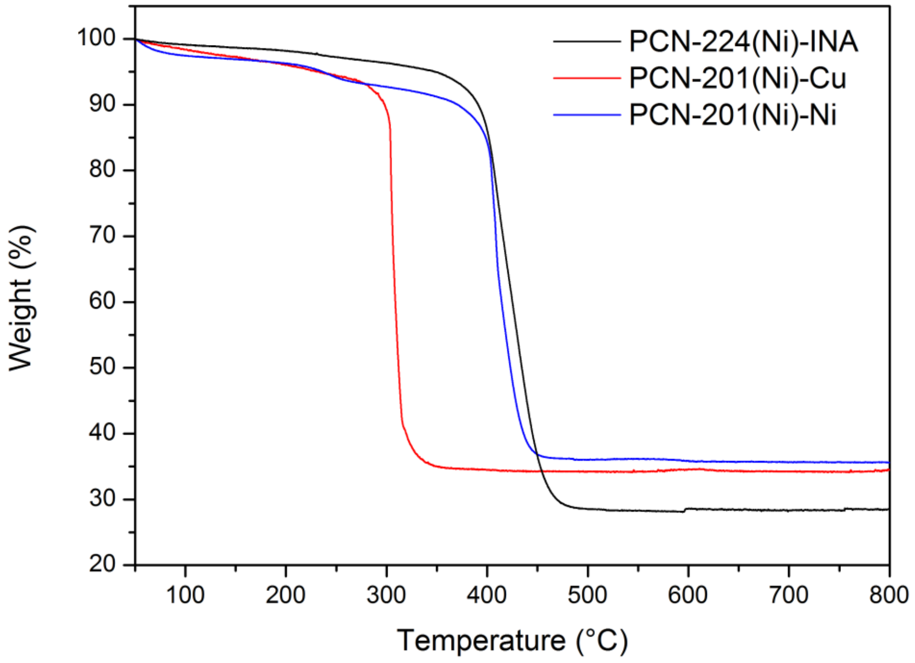

Supplementary Figure 14. Thermogravimetric analyses (TGA) of PCN-224(Ni)-INA, PCN-201(Ni)-Cu and PCN-201(Ni)-Ni.

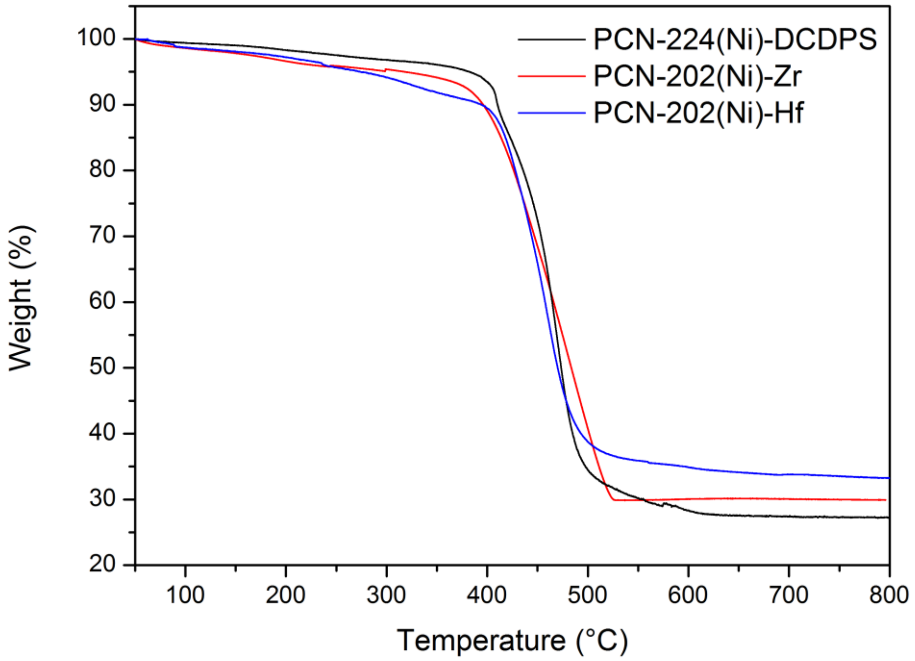

Supplementary Figure 15. Thermogravimetric analyses of PCN-224(Ni)-DCDPS, PCN-202(Ni)-Hf and PCN-202(Ni)-Zr.

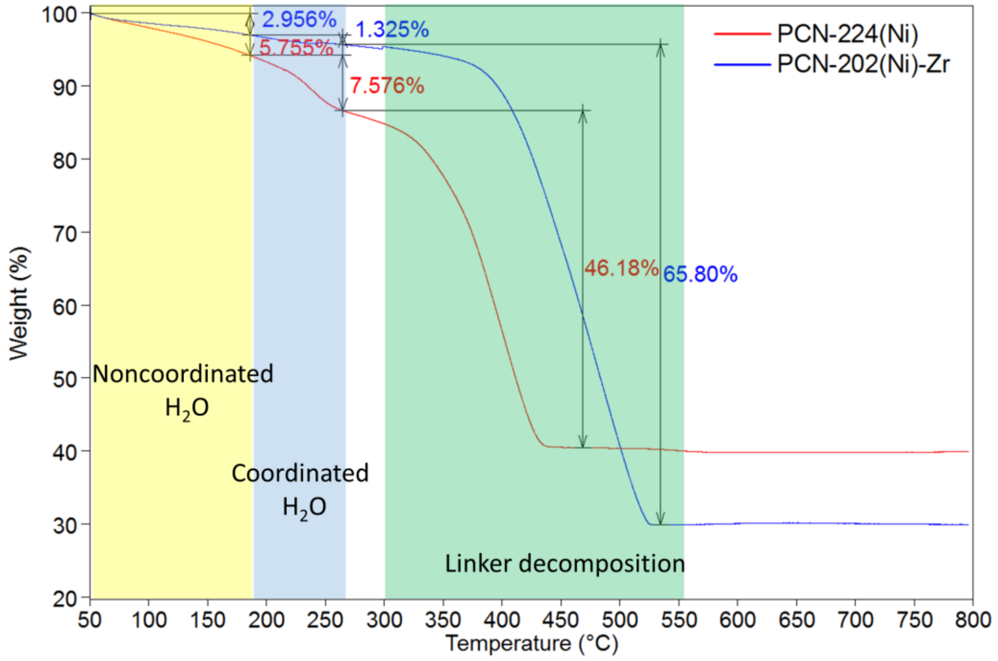

Supplementary Figure 16. A comparison of TGA curves for PCN-224(Ni) and PCN-202(Ni)-Zr.

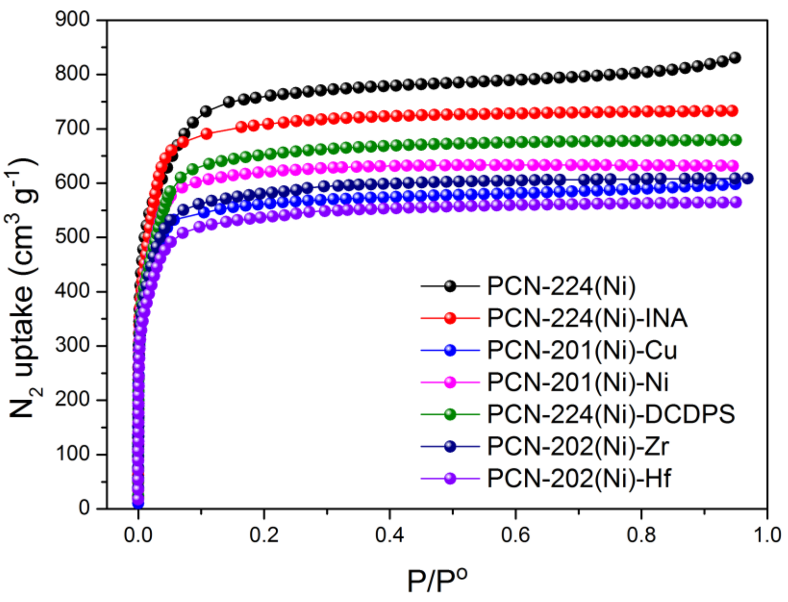

Supplementary Figure 17. N_2 adsorption isotherms of PCN-224(Ni), PCN-224(Ni)-INA, PCN-201(Ni)-Ni, PCN-201(Ni)-Cu, PCN-224(Ni)-DCDPS, PCN-202(Ni)-Zr, and PCN-202(Ni)-Hf at 77 K.

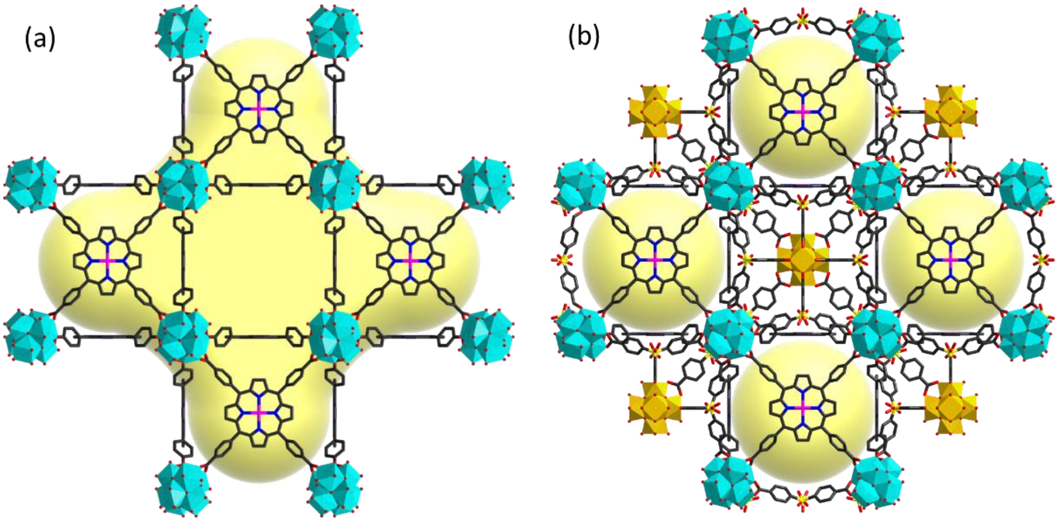

Supplementary Figure 18. The mesopores of PCN-224(Ni) are separated into micropores in PCN-202(Ni)-Zr as shown from single crystal structures.

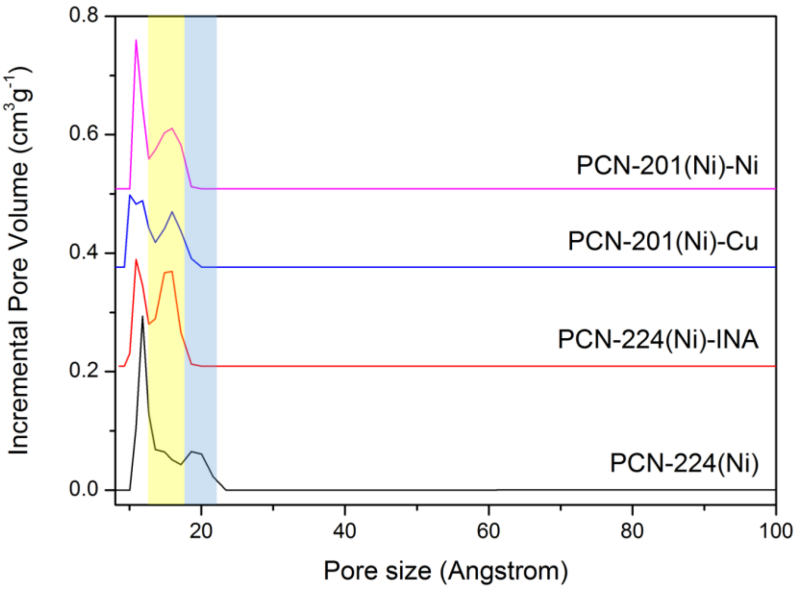

Supplementary Figure 19. DFT pore size distribution of PCN-224(Ni), PCN-224(Ni)-INA, PCN-201(Ni)-Ni, and PCN-201(Ni)-Cu calculated from N_2 isotherms at 77 K.

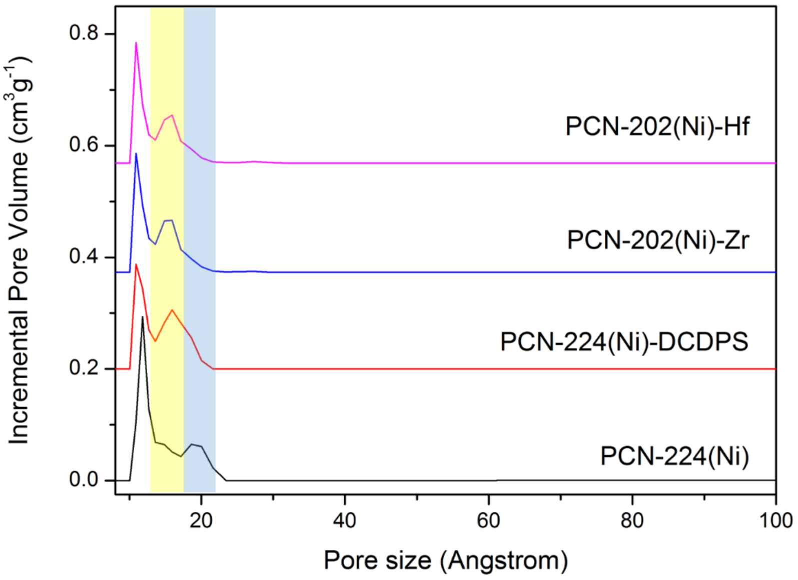

Supplementary Figure 20. DFT pore size distribution of PCN-224(Ni), PCN-224(Ni)-DCDPS, PCN-202(Ni)-Zr, and PCN-202(Ni)-Hf calculated from N_2 isotherms at 77 K.

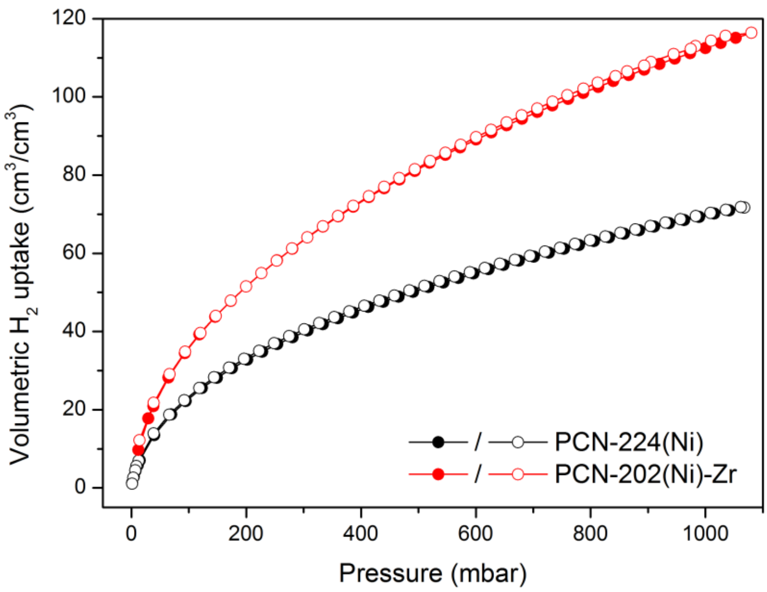

Supplementary Figure 21. Volumetric H_2 uptake of PCN-224(Ni) and PCN-202(Ni)-Zr at 77 K.

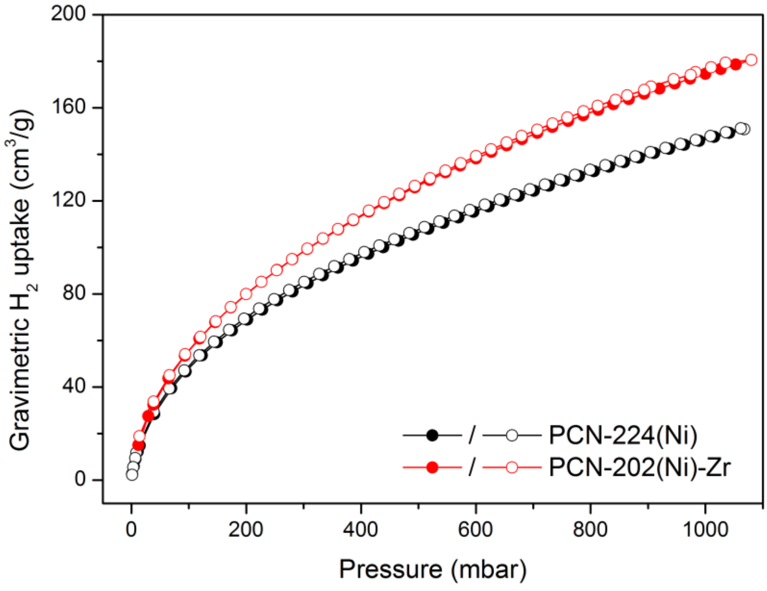

Supplementary Figure 22. Gravimetric H_2 uptake of PCN-224(Ni) and PCN-202(Ni)-Zr at 77 K.

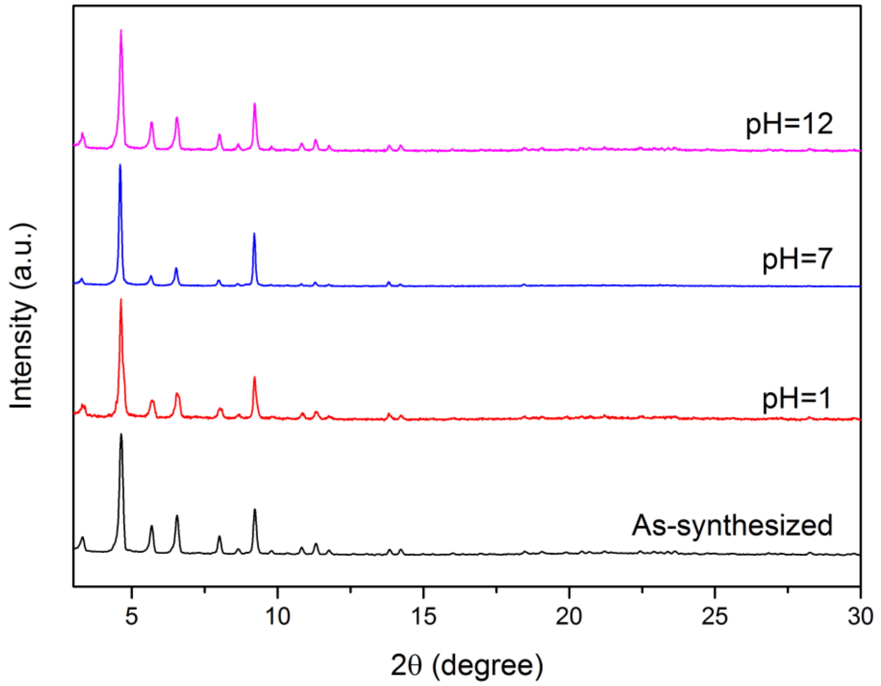

Supplementary Figure 23. PXRD patterns for PCN-202(Ni)-Zr in aqueous solution with pH ranging from 1 to 12 for 24 h.

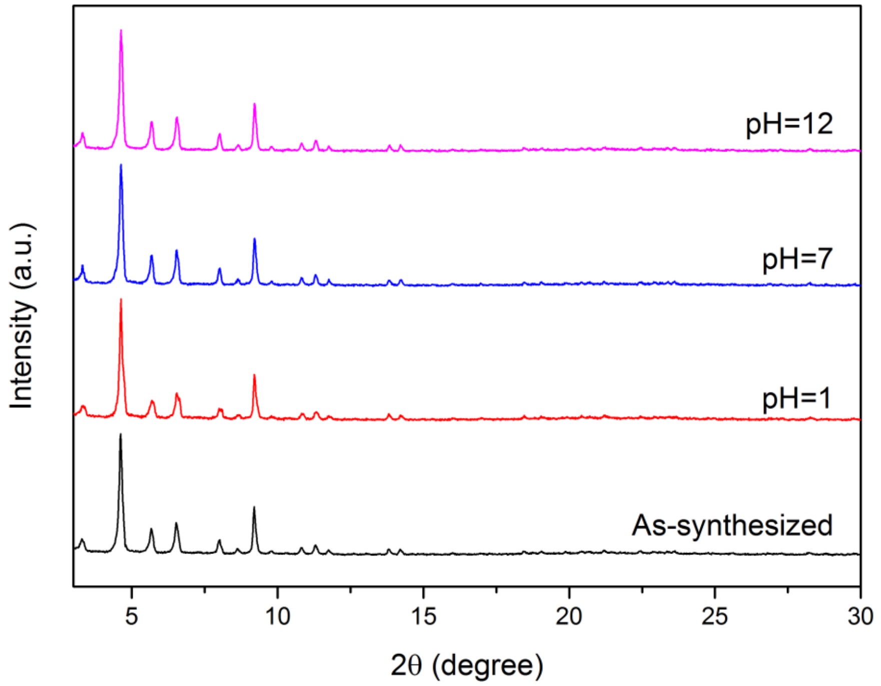

Supplementary Figure 24. PXRD patterns for PCN-202(Ni)-Hf in aqueous solution with pH ranging from 1 to 12 for 24 h.

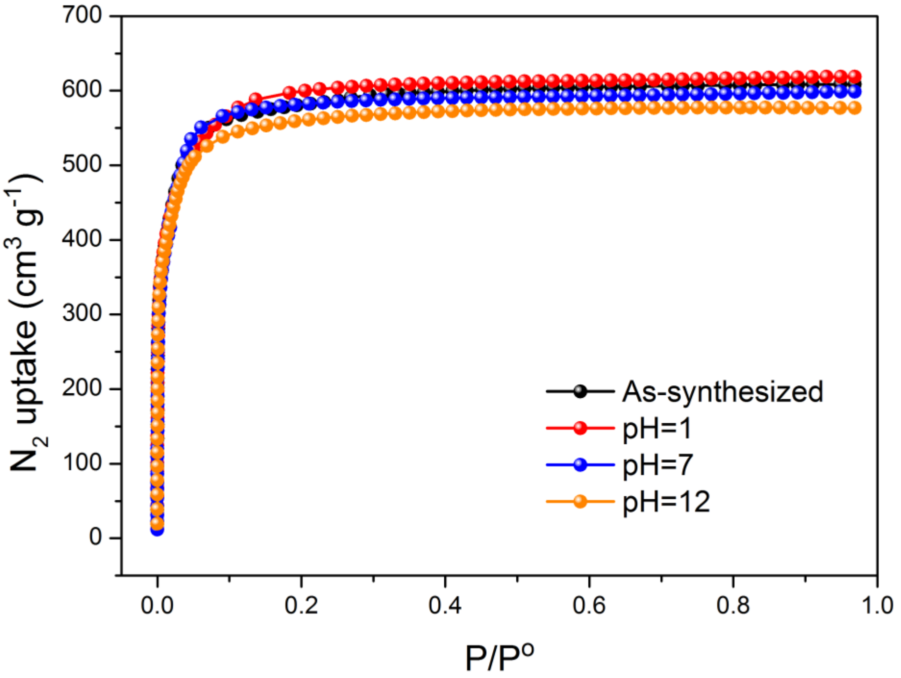

Supplementary Figure 25. N_2 adsorption isotherms of PCN-202(Ni)-Zr at 77 K after the treatment of aqueous solutions with pH ranging from 1 to 12 for 24 h.

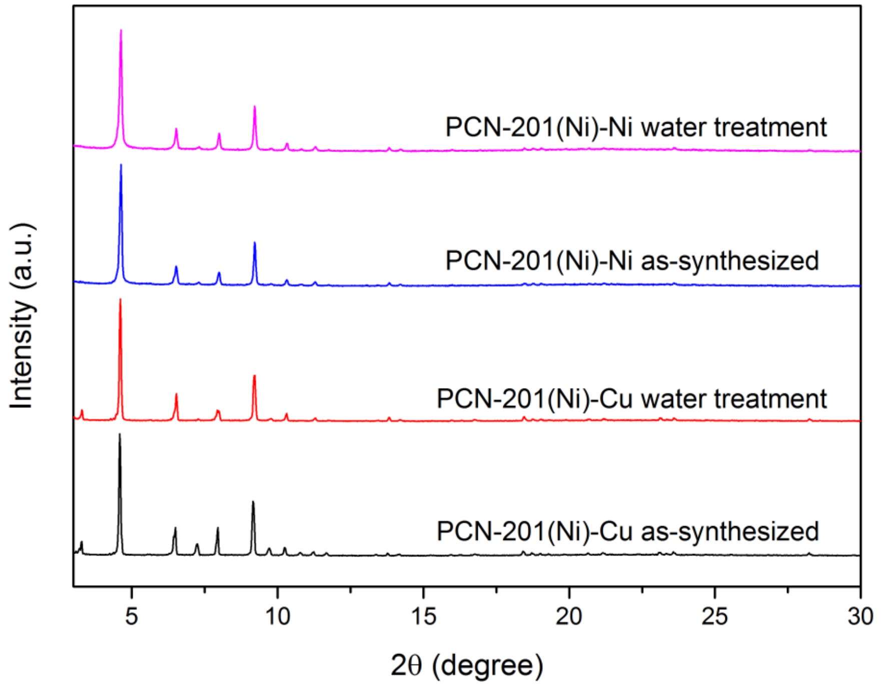

Supplementary Figure 26. PXRD patterns for PCN-201(Ni)-Ni and PCN-201(Ni)-Cu after water treatment for 24 h.

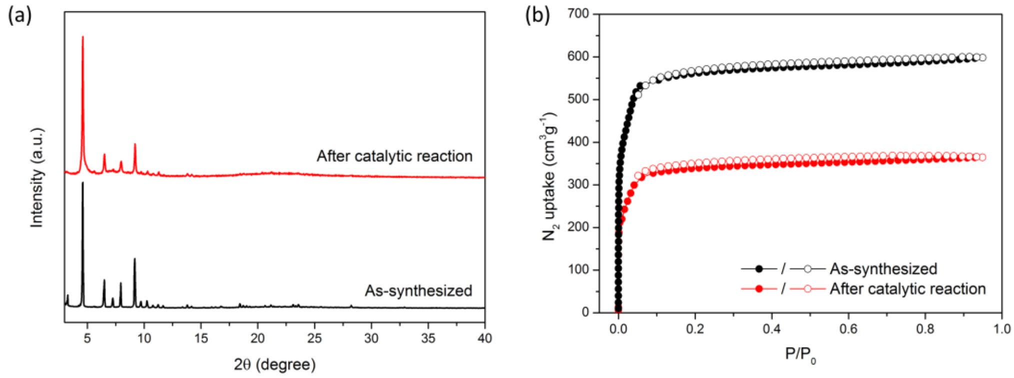

Supplementary Figure 27. (a) PXRD patterns and (b) N_2 sorption isotherms (77 K) for PCN-201(Fe)-Cu after catalytic reaction, indicating the maintained crystallinity and porosity.

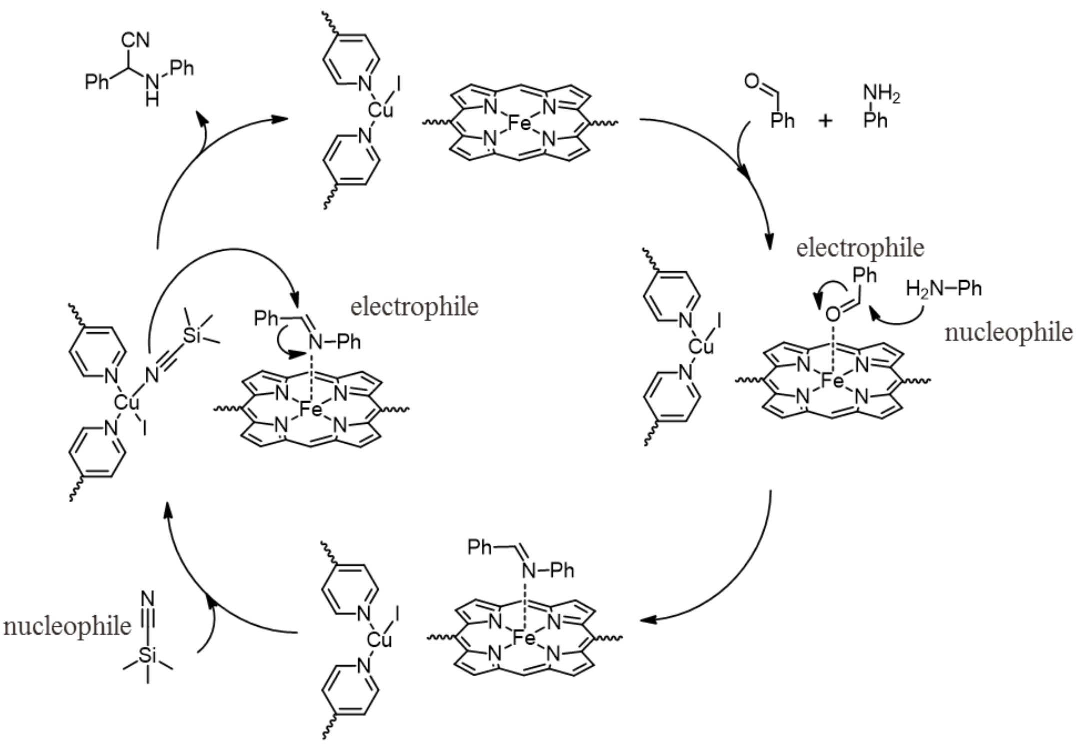

Supplementary Figure 28. Proposed mechanism for the three-component Strecker reaction catalyzed by PCN-201(Fe)-Cu.

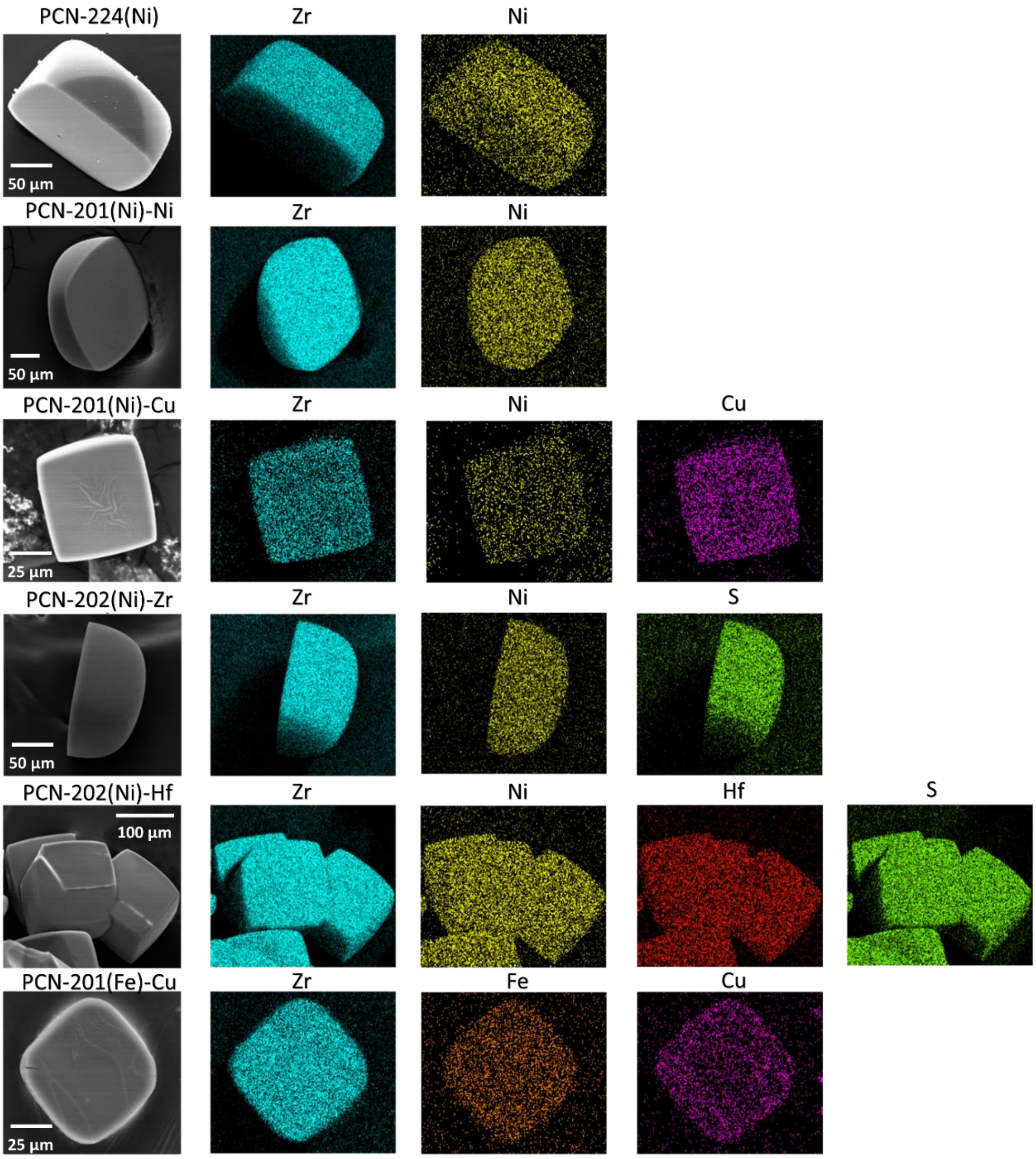

Supplementary Figure 29. SEM images and element mapping of PCN-224(Ni), PCN-201(Ni)-Ni, PCN-201(Ni)-Cu, PCN-202(Ni)-Zr, PCN-202(Ni)-Hf and PCN-201(Fe)-Cu.

Supplementary Table 1. Crystal data and structure refinements

Compound	**PCN-201 (Ni)-Cu**	**PCN-201 (Ni)-Ni**	**PCN-202 (Ni)-Zr**	**PCN-202 (Ni)-Hf**	**PCN-224 (Ni)-INA**	**PCN-224 (Ni)-DCDPS**
CCDC	**1544109**	**1544110**	**1544113**	**1544112**	**1544108**	**1544111**
Empirical formula	$C_{144}H_{80}N_{16}O_{42.67}Cu_4I_4Ni_2Zr_8$	$C_{144}H_{80}N_{16}O_{42.67}Ni_6Cl_4Zr_8$	$C_{912}H_{480}N_{48}O_{320}S_{24}Ni_{12}Zr_{60}$	$C_{960}H_{480}N_{48}O_{304}S_{24}Ni_{12}Hf_{12}Zr_{48}$	$C_{864}H_{480}N_{96}O_{256}Ni_{12}Zr_{48}$	$C_{912}H_{480}N_{48}O_{304}S_{24}Ni_{12}Zr_{48}$
Formula weight	4325.84	3940.72	24176.57	25544.29	21384.49	22825.93
Temperature/K	173(2)	173(2)	173(2)	173(2)	173(2)	100(2)
Crystal system	cubic	cubic	cubic	cubic	cubic	cubic
Space group	*Im*-3*m*	*Im*-3*m*	*Im*-3*m*	*Im*-3*m*	*Im*-3*m*	*Im*-3*m*
a/Å	38.3126(7)	38.3070(10)	38.418(5)	38.3665(8)	38.2762(6)	38.364(10)
b/Å	38.3126	38.307	38.418	38.3665	38.2762	38.364
c/Å	38.3126	38.307	38.418	38.3665	38.2762	38.364
α/°	90	90	90	90	90	90
β/°	90	90	90	90	90	90
γ/°	90	90	90	90	90	90
Volume/$Å^3$	56237(3)	56213(4)	56705(20)	56475.1(13)	56077(3)	56462(43)
Z	6	6	1	1	1	1
ρ_{calc}/(g cm^{-3})	0.766	0.698	0.708	0.751	0.633	0.671
μ/mm^{-1}	4.965	2.611	0.420	3.374	2.147	2.362
F(000)	12608.0	11720.0	11968.0	12512.0	10640.0	11360.0
Radiation	CuKα (*λ* = 1.54184 Å)	CuKα (*λ* = 1.54184 Å)	MoKα (*λ* = 0.71073 Å)	CuKα (*λ* = 1.54184 Å)	CuKα (*λ* = 1.54184 Å)	CuKα (*λ* = 1.54184 Å)
2Θ range for data collection/°	5.65 to 149.232	5.652 to 137.724	4.24 to 54.976	5.642 to 149.372	5.656 to 135.388	5.642 to 148.862
Reflections collected	93526	47618	596037	137606	42607	143603
Independent reflections	5368 [R_{int}= 0.1027, R_{sigma}= 0.0352]	4826 [R_{int}= 0.0491, R_{sigma}= 0.0269]	6036 [R_{int}= 0.1519, R_{sigma}= 0.0257]	5379 [R_{int}= 0.0502, R_{sigma}= 0.0139]	4649 [R_{int}= 0.0403, R_{sigma}= 0.0240]	5370 [R_{int}= 0.1153, R_{sigma}= 0.0356]
Data/restraints/parameters	5368/85/151	4826/51/141	6036/83/174	5379/87/136	4649/11/112	5370/18/121
Goodness-of-fit on F^2	1.050	1.432	2.071	2.577	1.353	1.865
Final R indexes [I>=2σ (I)]	R_1= 0.0748, wR_2= 0.2131	R_1=0.1008, wR_2= 0.3002	R_1= 0.1510, wR_2= 0.3926	R_1= 0.1804, wR_2= 0.4467	R_1= 0.1054, wR_2= 0.2979	R_1= 0.1510, wR_2= 0.4063
Final R indexes [all data]	R_1= 0.0918, wR_2= 0.2400	R_1= 0.1177, wR_2= 0.3348	R_1= 0.2143, wR_2= 0.4882	R_1= 0.2021, wR_2= 0.5135	R_1= 0.1255, wR_2= 0.3308	R_1= 0.1937, wR_2= 0.4598
Largest diff. peak/hole / e $Å^{-3}$	0.81/-0.92	1.66/-0.86	4.77/-2.39	6.35/-5.66	2.00/-0.76	1.99/-1.02

Supplementary Table 2. Compositional analysis of PCN-224(Ni), PCN-201(Ni)-Cu, PCN-201(Ni)-Ni, PCN-202(Ni)-Hf, PCN-202(Ni)-Zr and PCN-201(Fe)-Cu by ICP-MS.

	Formula	Theoretical	Experimental
**PCN-224(Ni)**	$[Zr_6O_4(OH)_8(H_2O)_4]_4$ (Ni-TCPP)$_6$	Zr:Ni = 4:1	Zr:Ni = 3.7:1
**PCN-201(Ni)-Cu**	$[Zr_6O_4(OH)_4]_4(CuI)_{12}$ (Ni-TCPP)$_6$(INA)$_{24}$	Zr:Ni:Cu = 4:1:2	Zr:Ni:Cu = 3.7:1:1.4
**PCN-201(Ni)-Ni**	$[Zr_6O_4(OH)_4]_4(NiCl_2)_{12}$ (Ni-TCPP)$_6$(INA)$_{24}$	Zr:Ni = 4:3	Zr:Ni = 3.8:3
**PCN-202(Ni)-Hf**	$[Zr_6O_4(OH)_4]_4[Hf_6O_4(OH)_4]$ (Ni-TCPP)$_6$(DCDPS)$_{12}$(HDPDCS)$_6$(OH)$_6$(H$_2$O)$_6$	Zr:Ni:Hf = 4:1:1	Zr:Ni:Hf = 3.5:1:0.91
**PCN-202(Ni)-Zr**	$[Zr_6O_4(OH)_4]_4[Zr_6O_4(OH)_4]$ (Ni-TCPP)$_6$(DCDPS)$_{12}$(HDPDCS)$_6$(OH)$_6$(H$_2$O)$_6$	Zr:Ni = 5:1	Zr:Ni = 5.5:1
**PCN-201(Fe)-Cu**	$[Zr_6O_4(OH)_4]_4(CuI)_{12}$ (FeCl-TCPP)$_6$(INA)$_{24}$	Zr:Fe:Cu = 4:1:2	Zr:Fe:Cu = 3.8:0.7:2

Supplementary Table 3. Compositional analysis of PCN-224(Ni), PCN-201(Ni)-Cu, PCN-201(Ni)-Ni, PCN-202(Ni)-Hf, PCN-202(Ni)-Zr and PCN-201(Fe)-Cu by ^{1}H-NMR.

	Formula	Theoretical	Experimental
**PCN-224(Ni)**	$[Zr_6O_4(OH)_8(H_2O)_4]_4$ (Ni-TCPP)$_6$	—	—
**PCN-201(Ni)-Cu**	$[Zr_6O_4(OH)_4]_4(CuI)_{12}$ (Ni-TCPP)$_6$(INA)$_{24}$	TCPP:INA = 1:4	TCPP:INA = 1:4.00
**PCN-201(Ni)-Ni**	$[Zr_6O_4(OH)_4]_4(NiCl_2)_{12}$ (Ni-TCPP)$_6$(INA)$_{24}$	TCPP:INA = 1:4	TCPP:INA = 1:5.04
**PCN-202(Ni)-Hf**	$[Zr_6O_4(OH)_4]_4[Hf_6O_4(OH)_4]$ (Ni-TCPP)$_6$(DCDPS)$_{12}$(HDPDCS)$_6$(OH)$_6$(H$_2$O)$_6$	TCPP:DCDPS = 1:3	TCPP:DCDPS = 1:3.55
**PCN-202(Ni)-Zr**	$[Zr_6O_4(OH)_4]_4[Zr_6O_4(OH)_4]$ (Ni-TCPP)$_6$(DCDPS)$_{12}$(HDPDCS)$_6$(OH)$_6$(H$_2$O)$_6$	TCPP:DCDPS = 1:3	TCPP:DCDPS = 1:3.06
**PCN-201(Fe)-Cu**	$[Zr_6O_4(OH)_4]_4(CuI)_{12}$ (FeCl-TCPP)$_6$(INA)$_{24}$	TCPP:INA = 4:1	TCPP:INA = 4.81:1

Supplementary Table 4. Three-component Strecker reaction catalyzed by MOFs.[a]

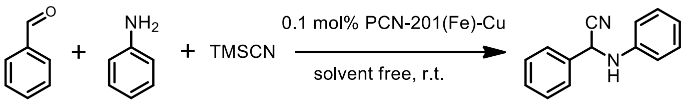

Entry	Catalyst	Substrate	Time (min)	Temprature (°C)	Yield (%)
**1**	PCN-201(Fe)-Cu	Benzyl aldehyde	10	25	99 ± 1[b]
**2**	PCN-201(Ni)-Cu	Benzyl aldehyde	10	25	21 ± 8[b]
**3**	PCN-224(Fe)	Benzyl aldehyde	10	25	24 ± 5[b]
**4**	PCN-201(Ni)-Cu and PCN-224(Fe) mixture	Benzyl aldehyde	10	25	68 ± 12[b]
**5**	PCN-201(Fe)-Cu	4-Methylbenzyl aldehyde	10	50	99
**6**	PCN-201(Fe)-Cu	4-Methoxybenzaldehyde	10	25	92
**7**	PCN-201(Fe)-Cu	4-Chlorobenzyl aldehyde	10	50	96
**8**	PCN-201(Fe)-Cu	4-Bromobenzyl aldehyde	10	50	82
**9**	PCN-201(Fe)-Cu	4-Cyanobenzyl aldehyde	10	50	81
**10**	PCN-201(Fe)-Cu	4-Nitrobenzyl aldehyde	10	50	68
**11**	PCN-201(Fe)-Cu	2-Furyl aldehyde	10	25	93
**12**	PCN-201(Fe)-Cu	Thiophene 2-carboxaldehyde	10	25	82
**13**	PCN-201(Fe)-Cu	Benzyl aldehyde	10	50	99
**14**	PCN-201(Fe)-Cu	Benzyl aldehyde	60	25	99
**15**[c]	PCN-201(Fe)-Cu	Benzyl aldehyde	10	25	96

[a]Reaction conditions: Generally, aldehyde (1 mmol), aniline (1 mmol), TMSCN (1 mmol), and catalyst (0.1 mol% based on Cu or 0.05 mol% based on Fe) were placed in a 4 mL vial and stirred at room temperature for 10 min. Yields were determined by ^{1}H NMR analysis and calculated based on the ratios of product/(product + starting material). [b]Values are mean ± standard error of three repeated experiments. [c]Recycled for 3 times.

Supplementary Methods

Materials and Instrument. All commercial chemicals were used without further purification unless otherwise mentioned. Powder X-ray diffraction (PXRD) was carried out with a BRUKER D8-Focus Bragg−Brentano X-ray powder diffractometer equipped with a Cu sealed tube (λ = 1.54178) at 40 kV and 40 mA. Single Crystal X-ray Diffraction (SC-XRD) was measured on a Bruker D8 Venture or D8 Quest diffractometer equipped with a Cu-$K\alpha$ (λ = 1.54184 Å, graphite monochromated) or Mo-$K\alpha$ sealed-tube X-ray source (λ = 0.71073 Å, graphite monochromated). Elemental analyses for C, H and N were carried out on a German Elementary Vario EL III instrument. Thermogravimetric analyses (TGA) were conducted on a TGA-50 (SHIMADZU) thermogravimetric analyzer. Gas sorption measurements were conducted using a Micrometritics ASAP 2420 system at various temperatures. NMR data were collected on a Mercury 300 spectrometer. Ultraviolet–visible absorption spectra were recorded on a Shimadzu UV-2450 spectrophotometer. ICP-MS data were collected with a Perkin Elmer NexION 300D ICP-MS. Field-emission SEM images were collected on the FEI Quanta 600 field-emission SEM (America) at 20 kV.

Single Crystal X-ray Crystallography. All crystals were taken from the mother liquid without further treatment, transferred to oil and mounted into a loop for single crystal X-ray data collection. Diffraction was measured on a Bruker D8 Venture or D8 Quest diffractometer equipped with a Cu-$K\alpha$ (λ = 1.54184 Å, graphite monochromated) or Mo-$K\alpha$ sealed-tube X-ray source (λ = 0.71073 Å, graphite monochromated). The raw frame data were processed using SAINT and SADABS to yield the reflection data file.[1] The structure was solved using the charge-flipping algorithm, as implemented in the program *SUPERFLIP*[2] and refined by full-matrix least-squares techniques against F_o^2 using the SHELXL program[3] through the OLEX2 interface.[4] The hydrogen atoms from the linkers were placed geometrically and refined using a riding model. The refinement of the framework was performed by ignoring the contribution of the disordered solvent molecules. The region containing the disordered electron density was identified by considering the van der Waals radii of the atoms constituting the ordered framework. Platon SQUEEZE[5] was used and .fab file was created containing partial structure factors representing the SQUEEZE region. The appropriate partial structure factors were used for input to SHELXL with the ABIN instruction. The ABIN instruction reads *h*, *k*, *l*, *A* and *B* from the file name fab, where *A* and *B* are the real and imaginary components of a partial structure factor.[3]

Refinement Details. For PCN201Cu, due to the weak diffractions at high Bragg angle, several atoms show unusual isotropic thermal parameters, thus several restraints were applied to ensure a reasonable refinement. In details, SIMU restraints

were also used for organic ligands and partial metal center with large thermal motion (O2 C9 C10 C12 N2 C11 C1 C2 O1 C4 C3 C6 C5 C7 C8 N1 Ni1). FLAT was used to ensure the planarity of some part of ligand. DFIX was used to fix the C9-C10 to 1.55 Å, C10-C11 and C11-C12 to 1.35 Å.

For PCN201Ni, due to the weak diffractions at high Bragg angle, several atoms show unusual isotropic thermal parameters, thus several restraints were applied to ensure a reasonable refinement. In details, SIMU restraints were also used for organic ligands with large thermal motion (O3 O4 O1 O2 C9 C10 C11 N2 C12 O1 C1 C2 C3 C4 C5 C6 C8 C7 N1). FLAT was used to ensure the planarity of some part of ligand (O2 C9 C10 C11 N2 C12). DFIX was used to fix the C12-C11, C0-C11 and N2-C12 to 1.35 Å, N2-C11 and C10-C12 to 2.35 Å, C9-C10 to 1.55 Å.

For PCN202Hf, due to the weak diffractions at high Bragg angle, several atoms show unusual isotropic thermal parameters, thus several restraints were applied to ensure a reasonable refinement. In details, SIMU restraints were also used for organic ligands with large thermal motion (O1 C1 C3 C2 C4 C6 C7 C8 C5 N4 O3 C9 C10 C11 C12 C13 S4 C14 C15 C16 C17 C18 O5). FLAT was used to ensure the planarity of some part of ligand (C10 C11 C12 C13 C9 and C13 C12_$1 C11_$1 C10 C11 C12 C9 S4, EQIV $1 +X,+Z,+Y). DFIX was used to fix the C1-O1, O5-C18, O3-C9 to 1.25 Å; C14-C15, C15-C16, C16-C17, C11-C12, C12-C13, C10-C11 to 1.35 Å; C15-C17, C14-C16, C11-C13, C10-C12 to 2.35 Å; C17-C18 and C9-C10 to 1.55 Å; C14-S4, C13-S4 to 1.85 Å; C10-O3 to 2.5 Å.

For PCN202Zr, due to the weak diffractions at high Bragg angle and not good crystal quality, several atoms show unusual isotropic thermal parameters, thus several restraints were applied to ensure a reasonable refinement. In details, SIMU restraints were also used for organic ligands with large thermal motion (S1 O1 O4 C9 > C18). FLAT was used to ensure the planarity of some part of ligand (S1 C9 C10 C11 C12 C13). DFIX was used to fix the C14-C15, C13-C12, C11-C12, C10-C11 and C9-C10 to 1.35 Å, C13-C11 and C10-C12 to 2.35 Å, S1-C12 to 2.70 Å.

For PCN224DCDPS, due to the weak diffractions at high Bragg angle and not good crystal quality, several atoms show unusual isotropic thermal parameters, thus several restraints were applied to ensure a reasonable refinement. In details, SIMU restraints were also used for organic ligands with large thermal motion (O4 S1 C10 C11 C12 C13 O5 C9). FLAT was used to ensure the planarity of some part of ligand (C10 C11 C12 C13 C9). DFIX was used to fix the C13-C12, C11-C12, C10-C11 to 1.35 Å, C9-C10 to 1.55 Å, C13-C11 and C10-C12 to 2.35 Å, C13-S1 to 1.9 Å, S1-O5 to 1.45 Å; O5-C13 to 2.6 Å.

For PCN224INA, due to the weak diffractions at high Bragg angle, several atoms show unusual isotropic thermal parameters, thus several restraints were applied to ensure a reasonable refinement. In details, SIMU restraints were also used for organic ligands with large thermal motion (N2 C12 C11 C10 C9 O2). FLAT was used to

ensure the planarity of some part of ligand (C10 N2 C12 C11). DFIX was used to fix the C12-C11, C10-C11, N2-C12 to 1.35 Å, N2-C11 and C10-C12 to 2.35 Å.

Topology Analysis. To better understand the structure of PCN-202(Ni)-Hf, topology analyses were carried out using TOPOS 4.0[3]. The disordered DCDPS linker appears as a "tritopic" linker because of the 2-fold disorder. Topologically, the 12-connected metal clusters can be regarded as cuboctahedron nodes and tetratopic TCPP linkers can be viewed as square nodes. If the 2-fold disordered DCDPS linker is regarded as a 3-connected triangle node, the overall structure can be simplified into a 3,4,12,12 connected net with a point symbol of $\{4^{24}.6^{36}.8^{6}\}_5\{4^{3}\}_{12}\{4^{4}.6^{2}\}_6$.

To eliminate the disorder of the DCDPS linker, we simulated an ordered structure of PCN-202(Ni)-Hf by reducing the space group from *Im*-3*m to I*-43*m*. The reduced space group with lower symmetry will eliminate the positional disorder of DCDPS linker by removing the mirror plan passing through its center. It should be noted that we attempted to refine the crystal structure of PCN-202(Ni)-Hf with a lower symmetry space group such as *I*-43*m*. However, the disorder is not eliminated, suggesting an inherent disorder of PCN-202(Ni)-Hf. In the simulated structure, each DCDPS linker is 2-connected to a Zr_6 cluster and a Hf_6 cluster. Therefore, each Zr_6 cluster is 9 connected to 6 TCPP and 3 DCDPS respectively, while each Hf_6 cluster is 12 connected to DCDPS linkers. Three pairs of DCDPS linkers bridges a pair of Zr_6 and Hf_6 cluster so that topologically they regarded as one edge. Consequently, Zr_6 clusters are simplified into a 5-connected hexagonal pyramid nodes while Hf_6 clusters are reduced into 4-connected tetrahedron nodes. The overall structure is simplified into a 4,4,7-connected net with point symbol of $\{4^{4}.6^{2}\}_6\{4^{6}.6^{15}\}_4\{6^{6}\}$. Note that the topology of ordered structure is dependent on the space groups that are chosen to eliminate the disorder. Different topologies might result if other space groups are selected to simplify the structure.

N_2 Sorption Isotherm. Before gas sorption experiments, as-synthesized sample was washed with *N*,*N*-dimethylmethanamide (DMF) and immersed in acetone for 3 days, during which the solvent was decanted and freshly replenished three times. The solvent was removed under vacuum at 100 ^{o}C, yielding porous material. Gas sorption measurements were then conducted using a Micrometritics ASAP 2020 system.

^{1}H NMR spectroscopy. For ^{1}H NMR spectroscopy of digested MOF samples, the activated samples (around 5 mg) were dissolved with saturated K_2CO_3 aqueous solution (1 mL), neutralize by 10 M HCl aqueous solution, and dried in a 100 ^{o}C oven. The solid was then dissolved in about 0.5 mL DMSO-d_6 for ^{1}H NMR analysis. For the catalytic reactions, the MOF catalyst was immediately separated from the reaction system after 10 min. About 100 μL of reaction supernatant was sampled, dissolved in 1 mL of dimethyl sulfoxide-d_6 and then measured by ^{1}H NMR. It took about 20 min before the spectrum was recorded.

SEM/EDX Analysis. Instrumental information of SEM/EDX: Images and analyses of

SEM/EDX were taken by FEI Quanta 600 FE-SEM. The Quanta 600 FEG is a field emission scanning electron microscope capable of generating and collecting high-resolution and low-vacuum images. It is equipped with a motorized x-y-z-tilt-rotate stage, providing the following movements: $x = y = 150$ mm (motorized); $z = 65$ mm (motorized); Tilt +70 degrees to –5 degrees (motorized); Source: Field emission gun assembly with Schottky emitter source. Voltage: 200 V to 30 kV. Beam Current: >100 nA.

ICP-MS Analysis. Samples were prepared in triplicate with weights of around 3 mg. Each sample was dissolved in J.T. Baker Ultrex® II Ultrapure 70% nitric acid at 70 ºC for 12 hours. Samples were then diluted to 150x in 1% nitric acid and 18.2 MΩ water from Millipore Milli-Q® water purification system. Calibration standards were prepared from certified reference standards from RICCA Chemical Company. Samples were further analyzed with a Perkin Elmer NexION® 300D ICP-MS. Resulting calibration curves have minimum $R^2 = 0.9999$. Additionally, in order to maintain accuracy, quality control samples from certified reference standards and internal standards were utilized. The individual results of the triplicate samples were averaged to determine the metal ratios.

Thermogravimetric Analysis. All the MOF samples were activated before thermogravimetric measurement. For thermogravimetric analysis, about 10 mg of the sample was heated on a TGA Q500 thermogravimetric analyzer from room temperature to 800 °C at a rate of 5 °C·min^{-1} under N_2 flow of 15 mL·min^{-1}. The result of thermogravimetric analyses for all the samples were shown in Supplementary Figures 14 and 15. The TGA curves of PCN-224(Ni) and PCN-202(Ni)-Zr were compared in Supplementary Figure 16. The initial weight loss before 185 °C (5.755% for PCN-224(Ni) and 2.956% for PCN-202(Ni)-Zr) is attributed to the removal of the water molecule in the pores. The weight loss from 185 °C to 265 °C is attributed to the removal of coordination water on the cluster, which is comparable to the theoretical mass percentage (calc. 7.244% for PCN-224(Ni) and 1.125% for PCN-202(Ni)-Zr). It should be pointed out that the terminal H_2O/OH^- groups on the Zr_6 clusters in PCN-224(Ni) is replaced by carboxylate groups from DCDPS linkers to form PCN-202(Ni)-Zr so that the H_2O content in PCN-224(Ni) is much higher than that in PCN-202(Ni)-Zr. This matched well with the experimental data. The decomposition of the framework starts at around 400 °C for PCN-224(Ni) and 450 °C for PCN-202(Ni)-Zr. The mass loss corresponding to the thermal decomposition of organics is 46.18% for PCN-224(Ni) and 65.80% for PCN-202(Ni)-Zr, which match well with the calculation (calc. 49.98% for PCN-224 and 68.51% for PCN-202(Ni)-Zr). PCN-202(Ni)-Zr shows higher thermal stability compared to PCN-224(Ni) possibly because of the higher connection number of Zr_6 clusters.

Single-crystal to Single-crystal Transformation Studies. To show that PCN-224

undergoes single crystal to single crystal transformation to generate PCN-202(Ni)-Hf, control experiments were conducted. The crystals of PCN-224(Ni) were observed by microscope during the modification process, which shows no change of crystal size or crystal shape (Supplementary Figure 1). The solution is colorless throughout the treatment of DCDPS and $HfCl_4$, indicating no dissolution of PCN-224(Ni). For comparison, the DMF solution containing 1 mg TCPP has a dark red color. The supernatant was separated and analyzed by UV, which eliminate the existence of TCPP (Supplementary Figure 3). The microscopic images of crystals for PCN-224(Ni), PCN-201(Ni)-Cu, PCN-201(Ni)-Ni, PCN-202(Ni)-Hf, PCN-202(Ni)-Zr, and PCN-201(Fe)-Cu were also compared in Supplementary Figure 2 which show almost identical size. These experimental results confirm that the formation of PCN-202(Ni)-Hf from PCN-224(Ni) is a single crystal to single crystal transformation process instead of a dissolution-recrystallization process.

Supplementary References

1. *APEX3*, *SAINT* and *SADABS*. Bruker AXS Inc., Madison, Wisconsin, USA, 2015.
2. Palatinus, L.; Chapuis, G. *J. Appl. Crystallogr.* 2007, **40**, 786.
3. Sheldrick, G. M. *Acta. Crystallogr., Sect. C* 2015, **71**, 3.
4. O. V. Dolomanov, L. J. Bourhis, R. J. Gildea, J. A. K. Howard, H. Puschmann, *J. Appl. Crystallogr.* 2009, **42**, 339.
5. Spek, A. L.; *Acta. Crystallogr., Sect. D*. 2009, **65**, 148.
6. Blatov, V. A. T. P., *Commission on Crystallographic Computing*, IUCr, 2006.
